# Incidence and Risk Factors of Retinopathy of Prematurity in Southeastern Iran: A Retrospective Cohort Study

**DOI:** 10.34172/aim.31070

**Published:** 2024-12-01

**Authors:** Alireza Maleki, Meisam Sargazi, Ali Yousefian, Saeedeh Sarhadi, Maryam Mollaei, Seyed Omid Mohammadi

**Affiliations:** ^1^Alzahra Eye Hospital Research Center, Zahedan University of Medical Sciences, Zahedan, Iran; ^2^Department of Ophthalmology, Faculty of Medicine, Mazandaran University of Medical Sciences, Sari, Iran; ^3^Department of Community Medicine, School of Medicine, Zahedan University of Medical Sciences, Zahedan, Iran; ^4^Health Promotion Research Center, Zahedan University of Medical Sciences, Zahedan, Iran; ^5^Anne Burnett Marion School of Medicine, Texas Christian University, Fort Worth, TX, USA

**Keywords:** Bevacizumab, Phototherapy, Retinal neovascularization, Retinopathy of prematurity, Risk factors

## Abstract

**Background:**

Retinopathy of prematurity (ROP) is a significant cause of preventable premature infant vision loss. Many studies have reported different risk factors depending on the region. In this study, we evaluated the incidence of ROP and associated risk factors in a referral eye hospital in southeastern Iran.

**Methods:**

This was a retrospective cohort study of preterm infants with birth weight (BW)≤2000 g or gestational age (GA)≤34 weeks and infants with GA between 34-37 weeks or GA<37 weeks and BW>2000 g referred by their pediatricians to our center from March 2022 until March 2023.

**Results:**

Totally, 920 infants met the inclusion criteria for the study. The incidence of ROP and ROP requiring treatment was 20.2% and 3.58% respectively. Results of logistic regression analysis showed higher GA and BW as protective factors against ROP occurrence. History of blood transfusion and phototherapy increased ROP development.

**Conclusion:**

Lower GA, lower BW, history of blood transfusion, and phototherapy were associated with ROP development. Phototherapy decreased the need for treatment among ROP patients.

## Introduction

 Retinopathy of prematurity (ROP) is a significant cause of preventable premature infant vision loss. The incidence of this disease is increasing in middle-income countries due to advances in prenatal and neonatal care.^1,2^ When infants are born prematurely, the oxygen levels are increased in the extra-uterine milieu compared to *in utero*. This relative increase leads to constriction of growing vessels. Subsequently, vascular obliteration ensues and retinal ischemia causes vessel endothelial growth factor upregulation. This leads to aberrant neovascularization and retinal detachment.^3-6^ Many studies have reported different risk factors depending on the region. Lower gestational age (GA) and lower birth weight (BW) are the two major risk factors for ROP development. Other risk factors associated with this condition include oxygen supplementation, multiple pregnancy, sepsis, history of blood transfusion, and intraventricular hemorrhage (IVH).^7-11^ Lower BW, lower GA, pulmonary disease, and number of blood transfusions were associated with ROP severity.^12,13^ Laser photocoagulation is the main treatment for ROP due to its safety and predictable outcomes. Intravitreal anti-vascular endothelial growth factors injection is another treatment option for the disease and promising results have been reported.^5,14,15^ There is no information available about the characteristics of ROP in Sistan and Baluchestan in southeastern Iran. In this study, we evaluated the incidence of ROP and its associated risk factors and provided a summary report of treatment results in a referral eye hospital.

## Materials and Methods

 This was a retrospective cohort study on 920 preterm infants referred to a referral eye hospital from March 2022 until March 2023.We includedall infants with BW ≤ 2000 g or GA ≤ 34 weeks and infants with GA between 34-37 weeks or GA < 37 weeks and BW > 2000 g referred by their pediatricians. Patients who did not complete follow-up examinations and had incomplete medical and informational records were excluded.

 The following data were collected and recorded: gender, BW, GA, number of pregnancies, oxygen therapy with continuous positive airway pressure (CPAP) or mechanical ventilation (number of days), history of blood transfusions, occurrence of IVH, occurrence of sepsis, phototherapy, mother’s age, type of delivery (cesarean section versus vaginal delivery), history of hypertension during pregnancy, history of pre-eclampsia, eclampsia, and hemolysis elevated liver enzyme levels and low platelet levels (HELLP) syndrome.

 The first examination was done between 4 and 6 weeks of chronological age or within the 31 and 33 weeks of postmenstrual age, whichever was later. Two experienced retina subspecialists examined infants after the instillation of tropicamide 0.5% for pupillary dilation. After topical anesthesia, fundoscopy was performed using an indirect ophthalmoscope and a ± 20 D or ± 30 D lens, an infant eyelid speculum, and a pediatric scleral depressor. The zones and stages of ROP were recorded based on the International Classification of Retinopathy of Prematurity, Third edition.^16^ The follow-up examination was done according to disease severity and continued until full retina vascularization.

 In our center, treatment is performed for patients categorized as type 1 ROP, aggressive ROP, and patients with stage 4 or stage 5 ROP.^16-18^ We treated ROP patients with type 1 ROP and aggressive ROP with intravitreal injection of bevacizumab 0.625 mg/0.025 mL. These patients were then re-examined on days 3 and 7 after injection and if the ROP stage, zone, and plus disease were improving, then we followed them every 1-2 weeks until the absence of neovascularization and improvement of plus disease. Then, we extended follow-up to every 3-4 weeks until 65 weeks. At this point, if retinal vascularization reached a border of less than 0.5 disc diameter away from the ora serrata in all quadrants, patients were considered to have “full retinal vascularization”, and no more follow-ups were scheduled.

 Retreatment with laser was done when there was sustained neovascularization and plus disease after initial therapy with intravitreal injection of bevacizumab, or when new extra-retinal neovascularization or development of plus disease occurred after the initial response to treatment as mentioned above. We treated patients when full retinal vascularization was not achieved at 65 weeks, with laser photocoagulation.

###  Statistical Analysis

 Sample size was calculated based on the previous ROP incidence rate of 40 % (19), type 1 error of 0.05, and a margin of error of 3.2%. Using the sample size formula for population proportion, 900 eligible infants were estimated. Finally, during the period of study, 920 participants were enrolled.

 Normally distributed quantitative data were described using mean and standard deviation (SD). The median and interquartile range (IQR) values represented asymmetrically distributed variables. The Kolmogorov-Smirnov test investigated the state of the normal distribution of quantitative variables. ROP risk factors were compared using the Mann-Whitney U test, chi-square, and Fisher’s exact tests. Additionally, a comparative analysis of risk factors was performed for the data gathered from ROP patients. The Mann-Whitney U test, chi-square, and Fisher’s exact tests were used for these comparisons. We used Fisher’s exact tests when the chi-square requirement was not met in 2*2 tables. The expected frequencies for each cell should be at least 1, and for the majority (80%) of the cells, the expected frequencies should be at least 5. To show the strength of the relationship between the variables, the effect size was calculated. The effect size was expressed by z/√N for the Mann-Whitney U test. Effect size, r, was interpreted as r ≤ 0.3 representing a small effect, 0.3 < r < 0.5, representing a medium effect, and 0.5 ≤ r representing a large effect. Also, the odds ratio and confidence interval of 95% were used to express the intensity of the relationship regarding chi-square and Fisher exact tests. An odds ratio greater than one meant increased odds of occurrence of ROP or treated ROP, and on the other hand, an odds ratio less than one was interpreted as lower odds of ROP or treated ROP compared to their non-occurrence. Finally, logistic regression analysis with the “Enter” method was used to determine the variables predicting the occurrence of ROP and ROP requiring treatment. Variables that were statistically significant in the univariate analysis were included in the regression analysis. In all comparison groups, the threshold of significance was 0.05. IBM SPSS Statistics for Windows version 27.0 (IBM Corp. 2013. Armonk, NY: IBM Corp) was used to perform the analyses.

## Results

 A total of 1143 newborn babies were referred to our hospital and screened for ROP. Of these, 920 infants met the inclusion criteria for the study. A total of 172 infants did not complete follow-up visits and 51 infants had incomplete medical records. Of 920 infants who met the inclusion criteria, 50.3% were male [n: 463]. The mean GA was 32.71 ± 2.11 weeks. Mean BW was 1809.45 ± 762.41 g.

 Of all screened infants, 561 infants received oxygen by CPAP, 56 infants were under mechanical ventilation, 51 infants received red blood cell transfusion, five infants had a history of IVH, and 25 infants developed sepsis. Also, 301 infants were the result of multiple pregnancy.

 The evaluation of risk factors and their relationship with ROP development and ROP needing treatment occurrence is shown in Table 1 and Table 2, respectively.

**Table 1:** Comparison of Risk Factors of Retinopathy of Prematurity (ROP) Between ROP and Without ROP Groups.

**Variable**		**Total (N=920)**	**ROP (n=186)**	**Without ROP (n=734)**	***P *** **Value**	**Effect Size**
Gestational Age (wk), Median (IQR)		33.0 (32,34)	30.0 (29,32)	33.0 (32,34)	< 0.001^a^	0.48^b^
Birth weight (g), Median (IQR)		1800 (1500,2000)	1425.0 (1195,1720)	1900.0 (1628,2100)	< 0.001^a^	0.41^b^
Gender, Male (%)		463 (50.3%)	95 (51.1%)	368 (50.1%)	0.81^c^	0.96 (0.69-1.32)^d^
Multiple births		301 (32.7%)	57 (30.6%)	244 (33.2%)	0.5^c^	0.88 (0.62-1.25)
CPAP, N (%)		561 (61%)	156 (83.9%)	405 (55.2%)	< 0.001^c^	4.22 (2.78-6.40)^d^
Days on CPAP		1.18 (1.8) 1.00 (2.00)	1.83 (2.70) 1.00 (1.44)	1.02 (1.44) 1.00(1.0)	< 0.001^a^	0.15
Mechanical ventilation, N (%)		56 (6.1%)	23 (12.4%)	33 (4.5%)	< 0.001^c^	2.99 (1.71-5.24)^d^
Days on mechanical ventilation		0.10 (0.54) 0.00 (0.00)	0.14 (0.67) 0.00 (0.00)	0.09 (0.5) 0.00 (0.00)	0.053 ^a^	0.06
Blood transfusion, N (%)		51 (5.5%)	36 (19.4%)	15 (2%)	< 0.001^c^	11.5 (6.14-21.54)^d^
Intraventricular hemorrhage, N (%)		5 (0.5%)	3 (1.6%)	2 (0.3%)	0.059^e^	6.0 (0.99-36.1)^d^
Sepsis, N (%)		25 (2.7%)	14 (7.5%)	11 (1.5%)	< 0.001^c^	5.34 (2.38-11.9)^d^
Phototherapy, N (%)		684 (74.3%)	155 (83.3%)	529 (72.1%)	0.002^c^	1.93 (1.27-2.94)^d^
Type of delivery, C/S, N (%)		606 (65.9%)	104 (55.9%)	502 (68.4%)	0.001^c^	1.7 (1.22-2.37)^d^
*In vitro* fertilization (IVF), N (%)		73 (7.9%)	14 (7.5%)	59 (8%)	0.81^c^	0.93 (0.50-1.70)^d^
Cause of premature Birth, N (%)	Onset of labor pains	334 (36.3%)	77 (41.4%)	257 (35%)	0.09^c^	
	Rupture of the amniotic sac	373 (40.5%)	77 (41.4%)	296 (40.3%)		
	Hypertension	140 (15.2%)	24 (12.9%)	116 (15.8%)		
	Other (preeclampsia, eclampsia, doctor’s diagnosis, HELLP syndrome)	73 (7.9%)	8 (4.3%)	65 (8.9%)		
Age of mother (year), Median (IQR)		29.0 (22,33)	25.0 (21,32)	29.0 (23,34)	< 0.001^a^	0.10^b^

SD, standard deviation; IQR, interquartile range;CPAP, Continuous positive airway pressure;C/S, cesarean section. ^a^Mann-Whitney U test. ^b^Effect size measure for Mann-Whitney U analysiswith r = z/√N. ^c^Chi-square test. ^d^Odds ratio (95% confidence interval). ^e^Fisher’s exact test.

**Table 2:** Comparison of Risk Factors of Retinopathy of Prematurity (ROP) Between ROP with and without Treatment Groups

**Variable**	**Total, N=186**	**Treatment** **Needed (n=33)**	**Without Treatment (n=153)**	***P *** **Value**	**Effect Size**
Gestational Age (wk), Median (IQR)	30.0 (29,32)	29.0 (27,30)	31.0 (29,32)	< 0.001^a^	0.35^b^
Birth weight (g), Median (IQR)	1425.0 (1195,1720)	1115.0 (1000,1315)	1500.0 (1255,1767.5)	< 0.001^a^	0.35^b^
Gender, Male, N (%)	95 (51.1%)	14 (42.5%)	81 (52.9%)	0.27^c^	1.52 (0.71-3.26)^d^
Multiple births	57 (30.6%)	14 (42.4%)	43 (28.1)	0.106^c^	1.88 (0.86-4.09)
CPAP, N (%)	156 (83.9%)	27 (81.8%)	129 (84.3%)	0.72^c^	0.83 (0.31-2.24)^d^
Days on CPAP	1.8 (2.7) 1 (1.5)	1.96 (2.88) 1.00 (1.0)	1.81 (2.67) 1.00 (2.00)	0.45^a^	0.05
Mechanical ventilation, N (%)	23 (12.4%)	8 (24.2%)	15 (9.8%)	0.02^c^	2.94 (1.12-7.67)^d^
Days on mechanical ventilation	0.14 (0.67) 0.00 (0.00)	0.12 (0.33) 0.00 (0.00)	0.15 (0.73) 0.00 (0.00)	0.55^a^	0.04
Blood transfusion, N (%)	36 (19.4%)	14 (42.4%)	22 (14.4%)	< 0.001^c^	4.38 (1.92-10.1)^d^
Intraventricular hemorrhage, N (%)	3 (1.6%)	1 (3%)	2 (1.3%)	0.44^e^	2.35 (0.20-26.8)^d^
Sepsis, N (%)	14 (7.5%)	4 (12.1%)	10 (6.5%)	0.27^c^	1.97 (0.57-6.72)^d^
Phototherapy, N (%)	155 (83.3%)	22 (66.7%)	133 (86.9%)	0.005^c^	0.30 (0.12-0.71)^d^
Type of delivery, C/S, N (%)	104 (55.9%)	21 (63.3%)	83 (54.2%)	0.32^c^	0.67 (0.31-1.47)^d^
*In vitro* fertilization (IVF), N (%)	14 (7.5%)	4 (12.1%)	10 (6.5%)	0.27^c^	1.97 (0.57-6.72)^d^
Cause of premature birth, N (%)					
Onset of labor pains	77 (41.4%)	16 (48.5%)	61 (39.9%)		
Rupture of the amniotic sac	77 (41.4%)	10 (30.3%)	67 (43.8%)	0.54^c^	
Hypertension	24 (12.9%)	5 (15.2%)	19 (12.4%)		
Other (preeclampsia, eclampsia, doctor’s diagnosis, HELLP syndrome)	8 (4.3%)	2 (6.1%)	6 (3.9%)		
Age of mother (year), Median (IQR)	25.0 (21,32)	25.0 (21,32)	24.0 (20,32)	0.70^a^	0.02^b^

SD, standard deviation; IQR, interquartile range;CPAP, Continuous positive airway pressure;C/S, cesarean section. ^a^Mann Whitney U test. ^b^effect size measure for Mann-Whitney U analysiswith r = z/√N. ^c^chi-square test. ^d^odds ratio (95% confidence interval). ^e^Fisher’s exact test.

 Univariate analysis showed statistically significant relationships between the occurrence of ROP and GA, BW, being under CPAP, the number of days under CPAP, being under mechanical ventilation, history of blood transfusion, history of sepsis, phototherapy, type of delivery (cesarean section), and lower maternal age (Table 1). The results of multivariate logistic regression analysis showed higher GA and BW as protective factors against ROP occurrence. History of blood transfusion and phototherapy increased ROP development. Additionally, phototherapy decreased ROP requiring treatment occurrence as shown in the logistic regression analysis in Table 3.

**Table 3:** Risk Factors for Retinopathy of Prematurity (ROP) and ROP Requiring Treatment Occurrence: Results of Logistic Regression Analysis

**Variables**	**Significance**	**Odds Ratio**	**95%CI**
Gestational Age	< 0.001	0.589	0.512-0.678
Birth weight	< 0.001	0.998	0.998-0.999
Blood transfusion	< 0.001	6.357	2.53-15.97
Phototherapy	0.006	2.172	1.24-3.78
Risk factors for ROP requiring treatment occurrence: results of logistic regression analysis			
Phototherapy	0.035	0.349	0.131-0.927

CI, confidence interval.

 A total of 186 infants had ROP and the incidence of ROP in our study was 20.2%. Twenty patients out of 33 ROP patients who needed treatment were in the ≤ 30 weeks GA group. There were 15 ROP patients in the ≤ 1000-g BW group and seven of them required treatment, although most ROP patients who needed treatment had 1000 < BW ≤ 1500 g (Table 4).

**Table 4:** Incidence of ROP and ROP Requiring Treatment in Different Gestational Age and Birth Weight Groups.

		**Total** ** N=920**	**ROP** **(n=186)**	**Without ROP** **(n=734)**	**ROP With Treatment ** **(n=33)**	**ROP Without Treatment (n=153)**
Gestational Age category (wk), No. (%)	< 34	172 (18.7%)	4 (2.2%)	168 (22.9%)	0 (0%)	4 (2.6%)
	32 < - ≤ 34	526 (57.2%)	56 (30.1%)	470 (64%)	2 (6.1%)	54 (35.3%)
	30 < - ≤ 32	151 (16.4%)	65 (34.9%)	86 (11.7%)	11 (33.3%)	54 (35.3%)
	≤ 30	71 (7.7%)	61 (32.8%)	10 (1.4%)	20 (60.6%)	41 (26.8%)
Birth weight category (g), No. (%)	< 2000	222 (24.1%)	9 (4.8%)	213 (29%)	0 (0%)	9 (5.9%)
	1500 < - ≤ 2000	499 (54.2%)	77 (41.1%)	422 (57.5%)	6 (18.2%)	71 (46.4%)
	1000 < - ≤ 1500	179 (19.5%)	85 (45.7%)	94 (12.8%)	20 (60.6%)	65 (42.5%)
	≤ 1000	20 (2.2%)	15 (8.1%)	5 (0.7%)	7 (21.2%)	8 (5.2%)

 Of the 33 patients treated in our center, 22 patients had type 1 ROP and 6 patients had aggressive ROP. We treated these 28 patients primarily with intravitreal bevacizumab (IVB) injection. Nine patients out of 28 patients needed laser retreatment. Five patients out of 33 patients had type 2 ROP and full retinal vascularization was not achieved at 65 weeks follow-up, so we treated them primarily with laser photocoagulation. There were no patients with stage 4 or stage 5 ROP during the period of our study.

## Discussion

 The incidence of ROP varies between different studies. Even in studies from various parts of Iran as shown in Table 5, screening programs, population heterogeneity, level of perinatal care, and NICU (neonatal intensive care unit) care may influence the statistical differences.^13^

**Table 5:** Incidence of ROP Occurrence in Different Studies

**Author** **Year of Publication** **Country **	**Study Design**	**Inclusion Criteria**	**Number of Patients**	**ROP Incidence (%)**	** Risk Factors**
Hu et al^19^ 2023 China	retrospective case–control study	BW < 1500 g	611	40.1%	Lower GA, twin birth, moderate to severe bronchopulmonary disorder
Bas et al^7^ 2018 Turkey	prospective cohort study	BW ≤ 1500 g or GA ≤ 32 weeks and those with a BW > 1500 g or GA > 32 weeks with an unstable clinical course	6115	27.0%	lower BW, smaller GA, total days on oxygen, late-onset sepsis, frequency of red blood cell transfusions and relative weight gain
Deb et al^20^ 2023 India	prospective cohort study	GA < 35 weeks and BW < 2500 gs	574	28.7%	lower BW, acyanotic heart disease, sepsis
Çömez et al^21^ 2022 Turkey	Retrospective Cohort study	GA > 31 ± 6 weeks to 36 ± 6 weeks	4156	22%	duration of hospital stay, BW and GA
Freitas et al^13^ 2018 Brazil	retrospective cohort study	GA l < 32 weeks or BW < 1500 g; or neonates born with 32-37 weeks' gestation or BW above 1500 g and any of the following associated: multiple gestation, respiratory distress syndrome, sepsis, blood transfusions or IVH	602	33.9%	extremely low BW, pulmonary diseases, IVH, and low GA
Al-Qahtani et al^10^ 2020 Saudi Arabia	retrospective cohort study	BW < 1501 g or GA < 32 weeks	581	38.6%	BW, GA, Postnatal steroid, O2 therapy, IVH, RBC transfusion
Dani et al^22^ 2021 Italy	cohort	GA between 23 and 30 weeks	178	38%	IVH, RBC transfusion
Zarei et al^23^ 2019 Iran	retrospective cohort study	GA ≤ 37 weeks	1,990	27.28%	GA, BW, and history of transfusion
Abrishami et al^9^ 2013 Iran	cross-sectional study	< 32 gestational weeks	122	26.2%	GA, sex, BW, Apgar score, duration of parenteral nutrition, oxygen therapy, phototherapy, maximum PaO2 and minimum SpO2 (univariate analysis)
Alizadeh et al^24^ 2015 Iran	cross-sectional study	BW ≤ 2500 g and/or GA ≤ 36 weeks	310	20.6%	low GA and low BW
Khorshidifar et al^25^ 2019 Iran	cross-sectional study	BW ≤ 2000 g or GA < 34 weeks and all other infants at risk of ROP admitted to the NICU or referred to our ROP clinic	207	33.3%	BW, GA and blood transfusion

GA, gestational age; BW, birth weight; IVH, intraventricular hemorrhage; NICU, neonatal intensive care unit; RBC, red blood cell.

 The incidence of ROP in our study was 20.2% among infants with BW ≤ 2000 g or GA ≤ 34 weeks or preterm infants with GA above 34 weeks or with BW above 2000 G with unstable clinical conditions referred by neonatologists.

 Totally, 185 patients with ROP had BW ≤ 2000-G or GA ≤ 34 weeks and only one patient did not fit in the previously mentioned inclusion criteria selected. This patient was directly referred for evaluation by a pediatrician.

 By implementing other criteria such as BW ≤ 1500 G or GA ≤ 32 weeks, BW ≤ 2000 G or GA ≤ 32 weeks, BW ≤ 1500 G or GA ≤ 34 weeks, 27 ROP patients, 6 ROP patients, and 4 ROP patients would be missed, respectively.

 The mean GA and BW of infants who developed ROP were 30.4 ± 2.1 weeks and 1447.8 ± 354.8 G, respectively. For ROP patients requiring treatment, the mean GA and BW were 28.84 ± 1.6 weeks and 1185.6 ± 294.8 G, respectively. The mean GA and BW for ROP requiring treatment in our study are within the range of GA 24-32 weeks and BW 700-1480 G previously reported by Khorshidifar et al in their study of ROP in Tehran, the capital of Iran.^25^

 Identifying risk factors for developing ROP helps set screening criteria, reduce unnecessary examinations, and may help prevent ROP occurrence or progression. Lower GA, lower BW, multiple births, red blood cell transfusions, phototherapy, IVH, supplemental oxygen therapy, sepsis occurrence, and pulmonary diseases are among the risk factors mentioned in the studies, as shown in Table 5.

 Like many other studies, lower BW and lower GA were correlated with ROP development.^7,9,10,13,21,23-25^

 Lower GA and lower BW suggest infants are more immature and are at risk for developing ROP due to general immaturity.^26^

 Blood transfusion was a risk factor for ROP development in our study. A lower concentration of HbF has been associated with ROP development.^27^ Blood transfusion replaces fetal hemoglobin (HbF) with adult hemoglobin (HbA) and may cause ROP development by increasing retinal oxygen delivery.^28,29^

 Phototherapy increased the risk of ROP development in our study. The antioxidant effects of bilirubin can be protective against ROP.^30^ However, in ROP patients, it reduced the need for treatment and was the only protective factor against ROP requiring treatment occurrence in our study.

 Gaton et al showed no protective role for bilirubin against ROP occurrence and no relation between ROP severity and bilirubin levels.^31^ However, a study by Boskabadi et al suggested that higher bilirubin levels may be protective against ROP development and may reduce ROP severity.^30^ It seems that the role of phototherapy as an independent factor needs further evaluation.

 Laser photocoagulation of avascular retina is the most common treatment for ROP, but it reduces the patient’s visual field significantly. In recent years, intravitreal anti-VEGF injections have been implemented. Although higher recurrence is reported in some studies, there is controversy between studies about the recurrence rate. Retinal tissue ablation, visual field reduction, and myopia occurrence are lower compared to laser photocoagulation.^5,15,32-34^

 In our study, the incidence of ROP requiring treatment was 3.58 %. A total of 28 patients received IVB injections as first treatment and 19 patients out of 28 patients achieved complete retinal vascularization during follow-up examinations. We followed up with the patients until 65 weeks of gestation and none of them showed recurrence after ROP regression.

 Nine out of 28 patients treated with IVB injection needed laser retreatment; six of them had aggressive ROP and did not respond well to primary IVB treatment, and three patients had type 1 ROP which showed incomplete vascularization until the 65-week follow-up visit.

 All patients who needed treatment in our study had GA ≤ 34 weeks and BW ≤ 2000 g. One limitation of our study is the retrospective design, which decreased our control on evaluations. The strength of our study is the large number of patients evaluated in our center.

## Conclusion

 The incidence of ROP occurrence in our study was 20.2%. Lower GA, lower BW, history of blood transfusion, and phototherapy were associated with ROP development. The incidence of ROP requiring treatment occurrence was 3.58% and phototherapy decreased the need for treatment among ROP patients.
